# High Performance NbMoTa–Al_2_O_3_ Multilayer Composite Structure Manufacturing by Laser Directed Energy Deposition

**DOI:** 10.3390/ma14071685

**Published:** 2021-03-30

**Authors:** Hang Zhang, Zihao Chen, Yaoyao He, Xin Guo, Qingyu Li, Shaokun Ji, Yizhen Zhao, Dichen Li

**Affiliations:** 1 State Key Laboratory for Manufacturing Systems Engineering, Xi’an Jiaotong University, Xi’an 710049, China; zhanghangmu@mail.xjtu.edu.cn (H.Z.); chenzihao18@foxmail.com (Z.C.); heyaoyao@stu.xjtu.edu.cn (Y.H.); jishaokun@stu.xjtu.edu.cn (S.J.); zyz8zyz@stu.xjtu.edu.cn (Y.Z.); dcli@mail.xjtu.edu.cn (D.L.); 2 State Key Laboratory for Strength and Vibration of Mechanical Structures, School of Aerospace Engineering, Xi’an Jiaotong University, Xi’an 710049, China; 3 Science and Technology on Reactor System Design Technology Laboratory, Nuclear Power Institute of China, Chengdu 610213, China; liqingyu9206@126.com

**Keywords:** laser additive manufacturing, metal–ceramic composite structures, MPEA, anisotropy

## Abstract

The conventional method of preparing metal–ceramic composite structures causes delamination and cracking defects due to differences in the composite structures’ properties, such as the coefficient of thermal expansion between metal and ceramic materials. Laser-directed energy deposition (LDED) technology has a unique advantage in that the composition of the materials can be changed during the forming process. This technique can overcome existing problems by forming composite structures. In this study, a multilayer composite structure was prepared using LDED technology, and different materials were deposited with their own appropriate process parameters. A layer of Al_2_O_3_ ceramic was deposited first, and then three layers of a NbMoTa multi-principal element alloy (MPEA) were deposited as a single composite structural unit. A specimen of the NbMoTa–Al_2_O_3_ multilayer composite structure, composed of multiple composite structural units, was formed on the upper surface of a φ20 mm × 60 mm cylinder. The wear resistance was improved by 55% compared to the NbMoTa. The resistivity was 1.55 × 10^−5^ Ω × m in the parallel forming direction and 1.29 × 10^−7^ Ω × m in the vertical forming direction. A new, electrically anisotropic material was successfully obtained, and this study provides experimental methods and data for the preparation of smart materials and new sensors.

## 1. Introduction

Metal–ceramic structures are multi-materials structures [1] that can provide enhancements to conventional alloys in terms of high-temperature strength, wear resistance, corrosion resistance, and hardness [2]. In addition, the fabrication of new multilayer metal–nonmetal composite structures provides unique solutions to many engineering problems [3]. Currently, the preparation of metal–ceramic structures is challenging due to various factors, such as the differences in the material properties of metals and ceramics and their inhomogeneous mixing; in some cases, ceramics only start to flow when the metal has evaporated [4], resulting in molded parts that are prone to delamination, cracking, uneven distribution, and other defects.

NbMoTa multi-principal element alloy, which have lattice distortion effects and cocktail effects [5,6,7], are unique and superior in performance compared with traditional alloys. They are usually prepared using the arc-melting method [8], so their size is limited by the size of the mold. The result is mostly button ingots, which limits their application to a small range of fields. Their high-temperature mechanical properties are excellent compared with those of conventional alloys [9].

Nowadays, additive manufacturing technology has attracted the attention of the manufacturing industry due to its advantages in forming various types of critical, complex structural parts in real life. It has broad application prospects in rapid tooling, rapid prototyping, composite parts, and direct parts manufacturing [10]. When some important influencing factors are improperly controlled, the products will have defects. Therefore, CT technology is introduced into the additive manufacturing process to evaluate metal and polymer additive manufacturing components, thereby reducing the number of trial-and-error experiments, shortening the time between design and production, reducing defects, and enabling more products to be cost-effective [11,12]. Laser-directed energy deposition (LDED) is a rapid additive manufacturing technique that uses a spherical powder as the raw material and a high-energy laser beam as the heat source to discretize the 3D model by layering it and then depositing it via point-by-point and layer-by-layer melting, according to the forming path [13]. On the one hand, this technique can be used to form NbMoTa and Al_2_O_3_ ceramics with a high energy density input from the laser beam [14]; on the other hand, this technique can be used to change components during the forming process by using its multiple powder feed bins, and has been used to fabricate composite structures of different kinds of metallic materials.

Kattire et al. [15] deposited CPM 9V on H13 substrates and found the presence of carbide–vanadium particles in the martensite and the residual austenite, which increased the hardness of the coating to four times the hardness of the matrix. Li et al. [16] prepared crack-free gradient Ti6Al4V/TiC composites with TiC volume fractions from 0 to 50% using a laser fusion deposition technique, and investigated the relationship between the microstructure and mechanical properties of the composites at different TiC volume fractions. Gualtieri et al. [17] prepared gradient composite structures of vanadium carbide and SS304, and found that the internal stress of the carbide significantly improved the hardness and wear resistance of the coatings.

Zhang et al. [4] successfully prepared Ti6Al4V, Ti6Al4V and Al_2_O_3_, Al_2_O_3_, and Ti6Al4V and Al_2_O_3_ composite structures using the directed energy deposition technique for the laser additive manufacturing of metal–ceramic composite structure designs. Sahasrabudhe et al. [18] prepared a CoCrMo–CaP composite structure. The addition of CaP made the face center cubic (FCC) and hexagonal close-packed (HCP) phases more stable and the microstructure showed a discontinuous chromium carbide phase, which improved the wear resistance. Metal–ceramic composites for prosthetic and biomedical applications have been studied [15,19,20], but research has not been done on the use of LDED technology to prepare MPEAs–ceramic composite structures.

This research aims to explore a new way to prepare NbMoTa–Al_2_O_3_ multilayer composite structures using LDED technology. It is intended to test and characterize the microstructure and properties of the NbMoTa–Al_2_O_3_ multilayer composite structure sample. Furthermore, this research plans to compare the hardness and wear resistance of the composite structure with NbMoTa.

## 2. Materials and Methods

**Figure 1:**
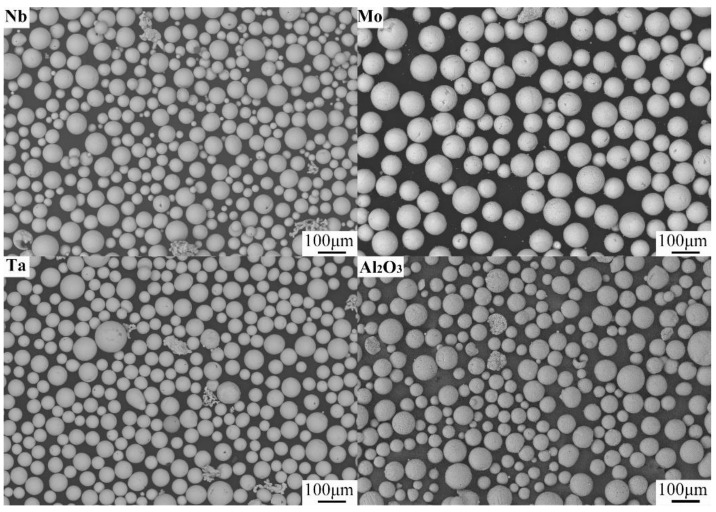
Nb, Mo, Ta, and Al_2_O_3_ powders.

Spherical Nb, Mo, and Ta powders with particle sizes of 45–75 microns and a purity of 99.9% were used as shown in Figure 1 to ensure that the powders had a suitable fluidity. The monolithic powders in equal molar proportions were weighed using electronic scales, mixed for 4h via SYH three-dimensional motion mixer series, and dried in a vacuum drying oven at 120 ℃ for 8 h before use.

**Figure 2:**
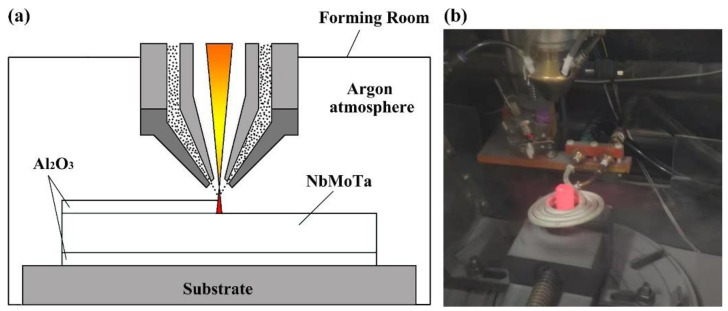
The forming process of the NbMoTa–Al_2_O_3_ multilayer composite structure. (**a**) A schematic diagram of the forming process; (**b**) an actual picture of the forming process.

The experiment was completed on the self-developed DED-1000B LDED system (Xi’an Jiaotong University, Xi’an, China), using an YLR-1000-MM-WC-Y11fiber laser (IPG Photonics Co., Oxford, MS, USA) with a working wavelength of 1064 nm and a spot diameter of 0.5 mm. Before the experiment, argon was used to fill the forming room as a protective gas to prevent the mixed powder from reacting with water and oxygen in the air at high temperatures. In addition, an induction heating pair was used to heat the cylinder to 600 °C. During the experiment, the water and oxygen content was kept within 80 ppm. The NbMoTa–Al_2_O_3_ multilayer composite structure specimen was formed on the surface of a φ20 mm × 60 mm cylinder. First, a layer of Al_2_O_3_ ceramic was deposited, and then three layers of NbMoTa was deposited, each of which serves as a structural unit of the composite structure. The deposition was carried out as shown in Figure 2. Before depositing the multilayer composite structure, single-factor experiments of power, scanning speed, powder-feeding rate, and other influencing factors were performed on the NbMoTa and Al_2_O_3_, and appropriate process parameters were selected according to the deposition effect. The depositing process parameters of NbMoTa and Al_2_O_3_ ceramic layers are shown in Table 1. The focal spot position is 0.6 mm below the sample surface.

After forming, the upper end of the cylinder was cut off by 1 mm using wire-cutting equipment, and the specimen was embedded in conductive resin, sanded by 400, 800, 1500 and 2500 grit sandpaper, polished by a polishing machine until the surface was bright and free of scratches, and then placed in an alcohol environment and cleaned by an ultrasonic cleaner for 3 min to remove the residual polishing solution impurities on the surface. The physical phase of the specimen was measured using D8 Advance A25 X-ray diffraction equipment (Bruker AXS GmbH, Karlsruhe, Germany), the microstructure of the longitudinal section of the specimen was observed using a Gemini SEM 500 scanning electron microscope (Cari ZEISS AG, Oberkochen, Germany) for composition testing, the microhardness was measured using an HXD-2000TMSC/LCD tester (Shanghai Tianming Optical Instrument Co., Ltd., Shanghai, China) with a load of 5 N and a dwell time of 15 s in the longitudinal direction, and the room temperature compression test was conducted by a CMT4304 multi-functional mechanical testing machine (MTS Systems (China) Co., Ltd., Shenzhen, China) at a strain rate of 0.001 s^−1^.

**Figure 3:**
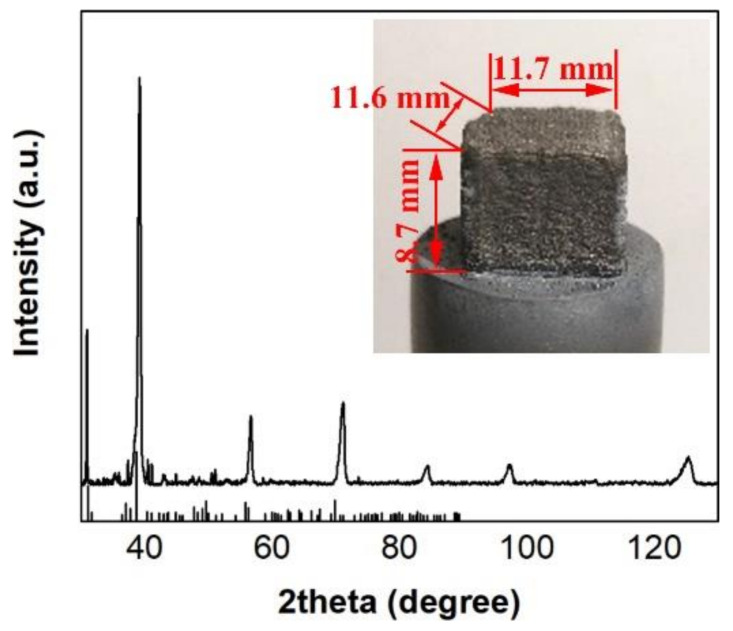
The X-ray diffraction (XRD) curve of the NbMoTa–Al_2_O_3_ multilayer composite structure specimen.

The tests were performed at 100 µm intervals, and three measurements were averaged for each height. The compressive strength in the parallel deposition direction was tested with a specimen size of 5 × 5 × 8 mm^3^. For the abrasion resistance test, a specimen with a size of 11.7 × 11.6 × 8.7 mm^3^, as shown in Figure 3, was prepared and rubbed 300 times using 180 grit sandpaper, glued to the projection lens in an up and down motion, and the weight change of the specimen before and after the test was measured using a VMS 553 wear resistance testing machine (Jia Teng Instrument Co., Ltd., Dongguan, China). The resistivities in the parallel and perpendicular molding directions were tested separately using the RTS-9 dual electric measuring four-probe tester (Four-Probe Technology Co., Ltd., Guangzhou, China) from Four-Probe Technology Co., Ltd.

## 3. Results and Discussion

### 3.1. Microstructure and Composition

Figure 3 shows the XRD curve of the NbMoTa–Al_2_O_3_ multilayer composite structure specimen. The analysis results show that the composite structure is a body-centered cubic (BCC) solid-solution structure. The addition of the Al_2_O_3_ ceramic caused the lattice distortion of NbMoTa, which increased the crystal plane distance of NbMoTa and caused a small displacement of the peak. A small amount of AlTaO_4_, which is a composite oxide formed by Al_2_O_3_ and Ta, may also be present.

**Figure 4:**
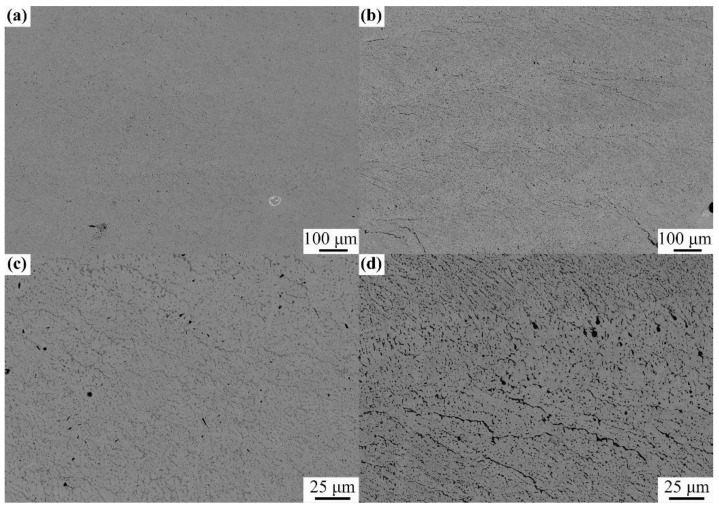
An scanning electron microscope (SEM) image of the composite structure. (**a**,**b**) multiple units at low magnification; (**c**,**d**) single unit at high magnification.

Figure 4 shows the microstructure images of the composite structure, and Figure 4a shows that the LDED effectively melted the powder under the process window of Table 1. The overall microstructure of the prepared composite structure was uniform, with fine equiaxed grains with an average grain size of 11.2 ± 5.4 µm, similar to the NbMoTa multi-principal element alloy prepared by Li et al. [21] using LDED, while the average grain size of the composite structure was smaller. This is because Al_2_O_3_ hinders the growth of NbMoTa grains, making the grain size of the composite structure finer than that of the NbMoTa multi-principal element alloy. With the multiple remelting caused by the continuous deposition of the composite structure, the precipitated phase is widely dispersed, further inhibiting the grain growth.

Figure 4b shows that there was no cracking or delamination between each composite structure consisting of one layer of Al_2_O_3_ and three layers of NbMoTa; therefore, this study has practical engineering significance. Figure 4c shows the microstructure within the same composite structural unit with a relatively uniform grain distribution and the presence of dark precipitated phases at the grain boundaries, mostly in the form of small particles dispersed on the grain boundaries. Figure 4d shows the junction region of the two composite structural units, where the precipitation phase at the junction is slightly increased, and a continuous curve-like precipitation phase with a scale of 100 µm appears, which indicates that more precipitation phases were generated during the deposition of the new composite structure.

**Figure 5:**
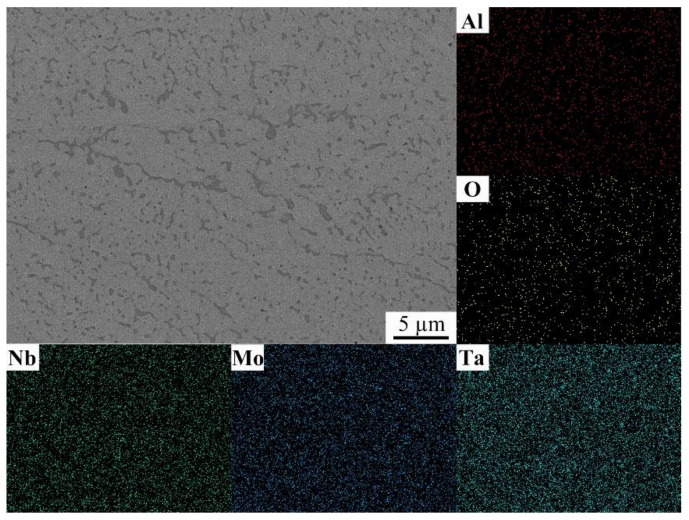
An EDS image of the NbMoTa–Al_2_O_3_ composite structure.

Figure 5 is the image obtained by an energy dispersive spectroscopy (EDS) image of the local part of Figure 4b, and a careful observation shows that Ta was uniformly distributed, Nb and Mo were less present in the precipitated phase, and Al and O were more present in the precipitated phase, which is consistent with the XRD diffraction image results, indicating that the precipitated phase may be AlTaO_4_.

**Figure 6:**
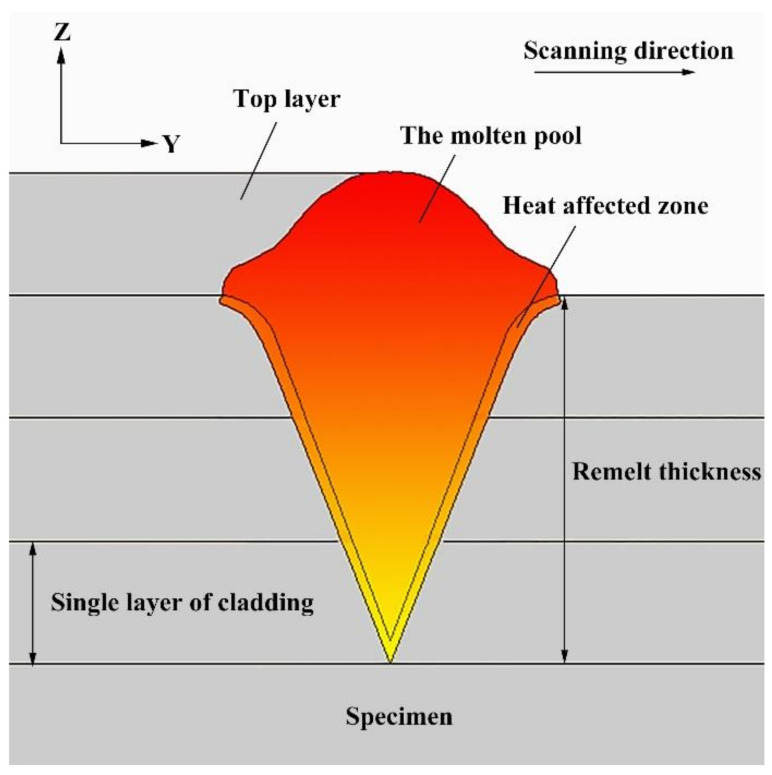
A schematic diagram of the remelting phenomenon.

The theoretical melting point of NbMoTa is 2750 °C, the melting point of Al_2_O_3_ is 2054 °C, and the boiling point is 2980 °C. Due to the prolonged reheating and diffusion time during LDED, the Al_2_O_3_ distribution in the continuously formed composite structure was uniform. The solidification rate of the melt pool remained the same, and the grain structure and morphology of the melt pool remained the same. When depositing NbMoTa, the temperature of the composite structure definitely exceeded the melting point of Al_2_O_3_, causing remelting of Al_2_O_3_, as shown in Figure 6. The remelting by the laser makes the existing Al_2_O_3_ become liquid, at which time the Nb, Mo, Ta, and Al_2_O_3_ materials are present inside the melt pool together, and this fluidity leads to a more adequate diffusion and makes it possible to form new substances from Nb, Mo, Ta, and Al_2_O_3_.

It is difficult to ascertain with complete certainty what the chemical formula of the precipitated phase on the grain boundaries is. An EDS analysis confirmed that the precipitated phase was mainly composed of Ta, Al, and O, and XRD analysis indicated that it may be AlTaO_4_. Therefore, there may be two different reasons for the existence of the precipitated phase on the grain boundaries. One is that the Al_2_O_3_ in NbMoTa may be completely saturated so that no more Al_2_O_3_ can be dissolved in the grain, and it therefore precipitates on the grain boundary because there is nowhere else for it to go; the other reason may be that, during the deposition of NbMoTa, Al_2_O_3_ remelts with Ta in a complex chemical reaction, eventually producing AlTaO_4_.

### 3.2. Mechanical Properties

**Figure 7:**
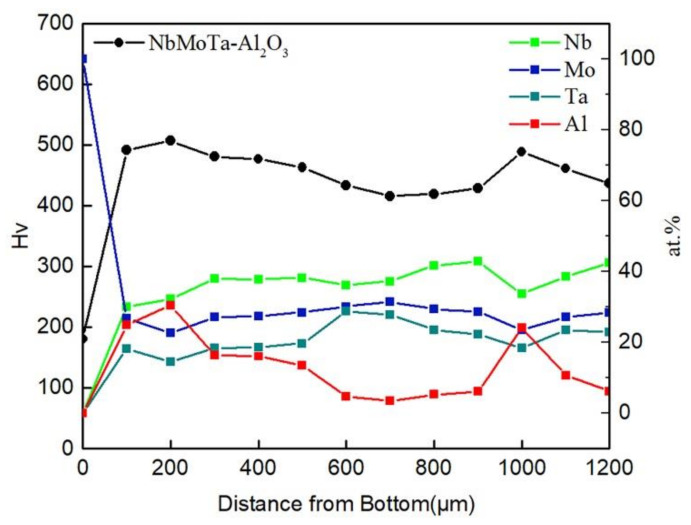
The hardness variation of the NbMoTa–Al_2_O_3_ composite structure.

The hardness of the longitudinal section of the NbMoTa–Al_2_O_3_ composite structure specimen was tested, and the average hardness variation with the deposition height is shown in Figure 7. The hardness of the composite structure was 462.5 ± 43.5 Hv_0.5_, and the trend of hardness variation is consistent with the trend of the Al content in the specimen. This is consistent with the analysis in Figure 4, and the change in hardness is due to the fact that the addition of Al_2_O_3_ makes the grain size of the composite structure finer and improves the hardness of the multilayer composite structure.

The high temperature gradient during LDED and the difference in the thermal expansion coefficients lead to residual stresses in the NbMoTa–Al_2_O_3_ composite structure, and the movement of the XRD peaks confirms the presence of lattice strain. The internal stresses in the NbMoTa–Al_2_O_3_ composite structure can also improve the hardness of the coating, and it has been shown that rapid solidification of the metal around the intermetallic reinforcement produces a strong matrix–particle bond [22,23] and contributes to load transfer across the phase interface, which forms the interfacial strengthening mechanism of the composite structure.

**Figure 8:**
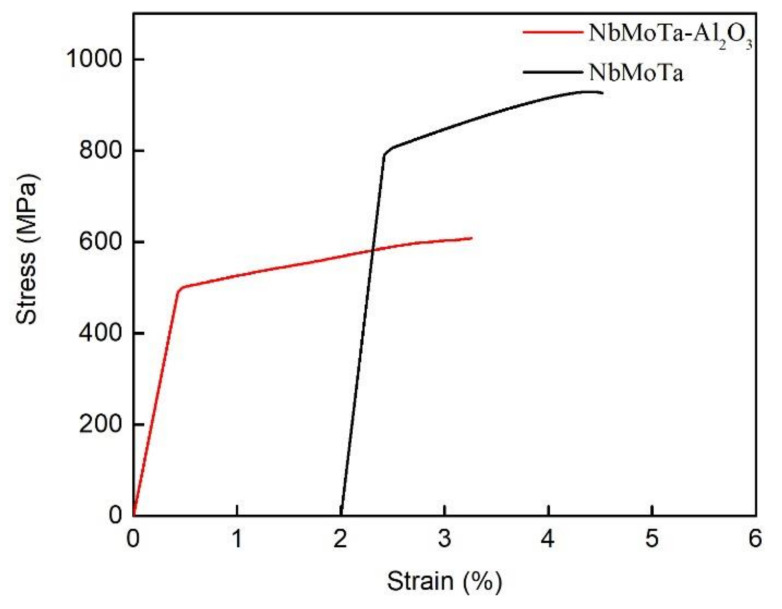
The compressive stress-strain curve of the NbMoTa–Al_2_O_3_ composite structure.

In addition, compression experiments were performed on the NbMoTa–Al_2_O_3_ composite structure specimens in the parallel deposition direction, and the results are shown in Figure 8. It can be seen that the yield strength of the composite structure was 502 MPa, the ultimate strength was 608 MPa, and the compression rate was 3.25%, while the yield strength of the NbMoTa prepared by LDED was 806 MPa, the ultimate strength was 929 MPa, and the compression rate was 2.5%. This is due to the presence of precipitated phases in the multilayer composite structure.

The precipitated phase plays a deflective and hindering role in crack extension, and, according to the confinement theory, the precipitated phase at the grain boundaries restricts the dislocation movement. The precipitated phase at the grain boundaries acts as a barrier to prevent or deflect cracks, increasing the effective mean path and allowing more pre deformation damage, which contributes to high-temperature strength [23].

**Figure 9:**
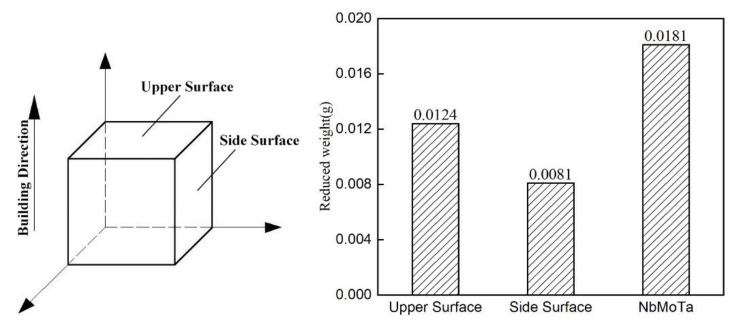
The loss of quality in abrasion resistance testing.

A 11.7 × 11.6 × 8.7 mm^3^ NbMoTa–Al_2_O_3_ composite structure square was deposited. The wear resistance of its upper surface and side was measured separately, and NbMoTa multi-principal alloys of the same size were formed for comparison. Using 180 grit sandpaper, we placed the square on the projection lens and rubbed it up and down 300 times (5 times/s with a pressure of 10 N), and then we measured the weight change in the sample before and after the test. The results are shown in Figure 9. The addition of Al_2_O_3_ ceramics significantly improved the wear resistance of NbMoTa, the surface mass loss on the NbMoTa–Al_2_O_3_ composite structure was reduced by 31.5%, and the side-surface mass loss was reduced by 55.3% compared to the NbMoTa multi-principal element alloy.

The precipitated phases at the grain boundaries increase the wear resistance by acting as abrasive particles. When wear occurs on a NbMoTa–Al_2_O_3_ multilayer composite structure specimen, the relatively soft NbMoTa starts to break down first, and the precipitated phases scattered at the grain boundaries have higher wear resistance and are protective of the worn specimen. Over time, the precipitated phase is excluded as debris and equates to abrasive particles when trapped in the contact area. The result is an increase in the mass loss due to the increase in abrasive particles.

The resistivity values of the specimens in the parallel forming direction and the perpendicular forming direction were tested. The resistivity of the specimens was 1.55 × 10^−5^ Ω × m in the parallel forming direction and 1.29 × 10^−7^ Ω × m in the perpendicular forming direction. This is because the overall direction of the precipitated phase has a certain angle with the forming direction, and the conductivity of the precipitated phase is poor. Therefore, electrons flow more easily in the parallel forming direction than in the vertical forming direction.

The ability to form composite structures by LDED offers significant advantages over adding Al_2_O_3_ powder directly to NbMoTa powder—the homogeneity of the material composition of the NbMoTa–Al_2_O_3_ composite structure reduces the likelihood of cracking and increases the interfacial strength. Compared to NbMoTa, Al_2_O_3_ is more brittle, and, under high loading, NbMoTa will deform more than Al_2_O_3_. This strain difference in conventional forming methods may lead to interlayer cracking of the metal and ceramic. By uniformly distributing Al_2_O_3_ in NbMoTa, the effect of strain differences is significantly reduced, thus reducing the likelihood of interlaminar cracking. This method can be used to increase the service life of many multi-principal alloy parts and is effective for parts operating in harsh environments.

## 4. Conclusions

This study confirms that LDED technology can be used to deposit two materials in cycles according to a specific layer ratio, and finally a multilayer composite structure is formed. This is a new solution for preparing composite structures. Through this research, we arrived at the following conclusions:

(1) The suitable layer distribution ratio of NbMoTa and Al_2_O_3_ was determined to be 3:1. The multilayer composite structure prepared by LDED has no macro cracks or holes. With the addition of Al_2_O_3_, the grain size of the NbMoTa–Al_2_O_3_ composite structure was smaller and more uniform than the NbMoTa multi-principal alloy;
(2) Precipitated phases exist on the grain boundaries, and the test results show that it may be that during the process of NbMoTa deposition, Ta and the remelted Al_2_O_3_ underwent a complex chemical reaction, and finally AlTaO_4_ was formed;
(3) Al_2_O_3_, as a strengthening particle, improved the wear resistance of the sample, which was higher than that of the NbMoTa multi-principal alloy formed by LDED. The resistivity of the specimens was 1.55 × 10^−5^ Ω × m in the parallel forming direction and 1.29 × 10^−7^ Ω × m in the perpendicular forming direction.

## Figures and Tables

**Table 1:** The depositing process parameters of different materials.

Materials	Laser Power (W)	Scanning Speed (mm/s)	*Z*-Axis Single Layer Travel (mm)	Overlap Rate (%)	Powder Mass Flows (g/min)	Volume Flow of Auxiliary Gas (L/min)	Volume Flow of ProTective Gas (L/min)
NbMoTa	550	8	0.08	50	2.96	5.0	4.0
Al_2_O_3_	200	8	0.08	50	1.47	4.0	4.0

## Data Availability

The data presented in this study are available in this article.
